# Patient characteristics, triage utilisation, level of care, and outcomes in an unselected adult patient population seen by the emergency medical services: a prospective observational study

**DOI:** 10.1186/s12873-020-0302-x

**Published:** 2020-01-30

**Authors:** Carl Magnusson, Johan Herlitz, Christer Axelsson

**Affiliations:** 1 Department of Molecular and Clinical Medicine, Institute of Medicine, Sahlgrenska Academy, University of Gothenburg, Gothenburg, Sweden; 2 Pre Hospen-Centre for Prehospital Research, Faculty of Caring Science, Work Life and Social Welfare, University of Borås, Borås, Sweden

**Keywords:** Patient safety, Emergency medical services, Triage, Patient assessment, Non-transport, Prehospital

## Abstract

**Background:**

Crowding in the emergency department (ED) is a safety concern, and pathways to bypass the ED have been introduced to reduce the time to definitive care. Conversely, a number of low-acuity patients in the ED could be assessed by the emergency medical services (EMS) as requiring a lower level of care. The limited access to primary care in Sweden leaves the EMS nurse to either assess the patient as requiring the ED or to stay at the scene. This study aimed to assess patient characteristics and evaluate the initial assessment by and utilisation of the ambulance triage system and the appropriateness of non-transport decisions.

**Methods:**

A prospective observational study including 6712 patients aged ≥16 years was conducted. The patient records with 72 h of follow-up for non-transported patients were reviewed. Outcomes of death, time-critical conditions, complications within 48 h and final hospital assessment were evaluated. The Mann-Whitney U test, Fisher’s exact test, and Spearman’s rank correlation were used for statistical analysis.

**Results:**

The median patient age was 66 years, and the most common medical history was a circulatory diagnosis. Males received a higher priority from dispatchers and were more frequently assessed at the scene as requiring hospital care. A total of 1312 patients (19.7%) were non-transported; a history of psychiatric disorders or no medical history was more commonly noted among these patients. Twelve (0.9%) of the 1312 patients not transported were later admitted with time-critical conditions. Full triage was applied in 77.4% of the cases, and older patients were triaged at the scene as an ‘unspecific condition’ more frequently than younger patients. Overall, the 30-day mortality was 4.1% (*n* = 274).

**Conclusions:**

Age, sex, medical history, and presentation all appear to influence the initial assessment. A number of patients transported to ED could be managed at a lower level of care. A small proportion of the non-transported patients were later diagnosed with a time-critical condition, warranting improved assessment tools at the scene and education of the personnel focusing on the elderly population. These results may be useful in addressing resource allocation issues aiming at increasing patient safety.

## Background

In Gothenburg, the second largest city in Sweden, the patient volumes in the emergency department (ED) and those treated by the emergency medical services (EMS) are increasing, as in many other national and international health-care systems [1–3]. Aiming to reduce the time to definitive care, EMS pathways have been introduced to bypass the ED for certain subgroups of patients, such as those with stroke, myocardial infarction, cardiac arrest, hip fractures, infections, or an assessed need for inpatient care. On the other hand, many ED presentations are regarded as low acuity, where other levels of care may be more appropriate [4–6]. Referral to primary care (PC) may be more beneficial for a significant number of these patients [7, 8]. In Sweden, all ambulances are staffed by at least one registered nurse. The EMS nurse has been given the responsibility to decide upon the most appropriate level of care. For low-acuity patients in Sweden, there is a lack of urgent care centres or geriatric centres as a level of care between the ED and PC. This leaves the EMS nurse with limited options: either to transport the patient to the ED, or to allow the patient to stay at the scene with or without a PC appointment, which may take place days or weeks later. Low-acuity ED presentations can result from this lack of access to PC for immediate care [9].

In the prehospital setting, a significant amount of preventable harm to patients is associated with clinical decision making [10]. To aid the EMS nurse in patient assessment, a mandatory triage system was introduced in 2010. The rapid emergency triage and treatment system for adults (RETTS-A) was initially developed for triage in the ED in order to stratify patients based on severity and physician waiting time. Studies of RETTS-A (ED) have reported that increased mortality and in-hospital stay were associated with a higher triage level, with an ED nurse inter-reliability of moderate to good [11, 12]. Studies of the Danish emergency process triage (DEPT), the Canadian triage and acuity scale (CTAS), and the emergency severity index (ESI) have reported only moderate agreement between EMS clinicians and ED nurses when utilising identical triage systems [13–15]. RETTS-A was not developed to be utilised as a system assessing whether low-acuity patients should stay at the scene with self-care or referral to PC. There is inconsistency in what characterises a non-transport patient, and various guidelines and policies are used in different EMS organisations. A study in Sweden of patients triaged to the lowest level (Green) according to RETTS-A reported a decrease in ED presentations when the EMS nurse consulted a PC physician on the most appropriate level of care for selected patients [16]. However, there is limited knowledge regarding the initial EMS nurse assessment of patients in contact with the emergency telephone number in Sweden and the utilisation of a triage system in an unselected EMS population.

Therefore, this study aimed to 1) describe the characteristics of the EMS population and evaluate the initial assessment by and utilisation of the RETTS-A, and 2) assess the appropriateness of non-transport decisions.

## Methods

### Study design

The present study is a single-centre prospective, observational study. All the EMS crews were informed about the study in weekly letters both before and during the study period. To increase data quality and conformity, workplace meetings were held before the commencement of the study, and the EMS crews were given repetition training in the triage system, including written instructions.

### Study setting

The EMS organisation operates in an urban area in the western part of Sweden covering approximately 900 km^2^, and serves a community of 660,000 inhabitants (as at the time of the study). The EMS receives assignments through a regional dispatch centre. Incoming calls are assessed with the aid of a dispatch medical index (DMI) and prioritised by level of urgency. Priority 1 is considered life threatening, priority 2 as urgent but not life threatening, priority 3 as no medical risk regarding waiting time, while priority 4 is assigned to patients who only need transport and is carried out by non-emergency transports staffed by one emergency medical technician. Annually, the EMS organisation exceeds 80,000 priority 1–3 assignments, of which 58,575 assignments are considered primary, where a patient assessment takes place. The EMS organisation operates with a differentiated fleet of 22 units during the day and 12 units during the night, including two nurse-staffed single responders, one physician-staffed unit, and one scene commanding unit. All ambulances in the organisation that respond to priority 1–3 assignments are advanced lifesaving (ALS) units. Within the EMS organisation, the majority of the registered nurses have a postgraduate education specialising in prehospital emergency care.

### Materials

**Fig. 1:**
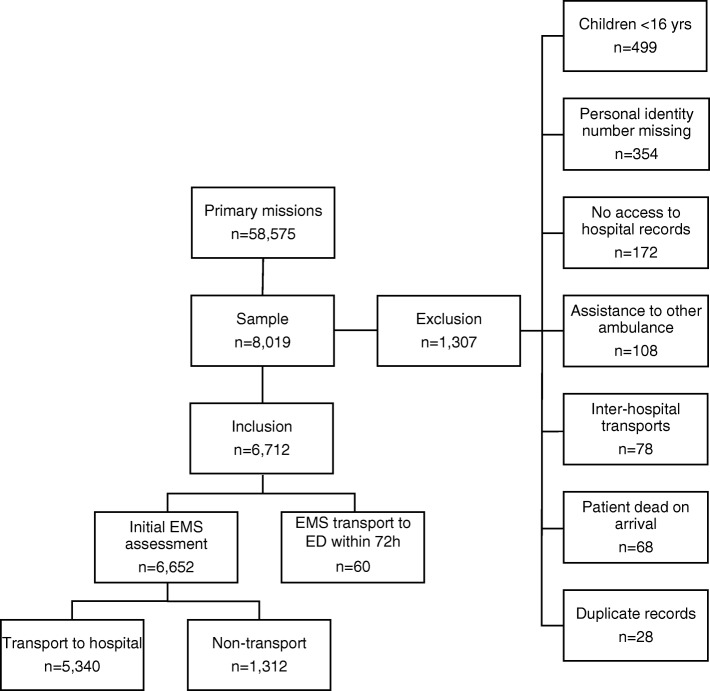
Flow chart of the studied patients, and the distribution of patient assessment

A consecutive sample was collected over the course of 1 year (2016) from the first 1000 assignments each month. The inclusion criterion was assignments in which a patient assessment by an EMS nurse took place (i.e., primary assignments). The exclusion criteria were: 1) patient age < 16 years, 2) inter-hospital transports, 3) assignments with no patient contact, 4) assistance to another EMS unit, and 5) dead on arrival. A total of 8019 assignments were initially included from the 12,000 eligible for inclusion in the study. After a full manual review of records, 1307 assignments were excluded, leaving 6712 (11.5% of annual primary assignments) assignments fulfilling the inclusion criterion. Of the 6712 included assignments, 6652 patients were identified as assignments with an initial EMS contact, and they are presented in Tables 1, 3, 4, and 5, while patients with a ED visit within 72 h are presented in Table 2 of which 60 patients had a secondary EMS assessment and transport to the ED and 66 patients were transported by own means to the ED. Data were collected from both EMS (Ambulink) and hospital records (Melior) (Fig. 1). Ambulink contains the RETTS-A triage classification and Melior contains the international classification of diagnosis (ICD) code. Medical history and final hospital assessment have been categorised using the ICD–10 chapters (2016). The ICD-10 is structured into 22 chapters I-XXII, for example chapter IX is Diseases of the circulatory system (I00-I99) that includes all diagnoses of the circulatory system such as hypertension, stroke or myocardial infarction.

**Table 1:** Total distribution of EMS assignments and characteristics with comparison of female and male

	Total	Female	Male	*P*^*1*^
	*n* = 6652	*n* = 3525	*n* = 3127	
Age – years (25th,75th percentiles)				
Median	66 (42,82)	69 (42,84)	64 (42,79)	< 0.001
Medical history^a^ – n (%)				
Diseases of the circulatory system I00-I99	6137 (28.6)	3121 (26.9)	3016 (30.7)	< 0.001
Mental and behavioural disorders F00-F99	3811 (17.8)	2056 (17.7)	1755 (17.8)	0.830
Endocrine, nutritional and metabolic diseases E00-E89	1906 (8.9)	1102 (9.5)	804 (8.2)	0.001
Diseases of the musculoskeletal system and connective tissue M00-M99	1449 (6.8)	1034 (8.9)	415 (4.2)	< 0.001
Diseases of the digestive system K00-K95	1225 (5.7)	713 (6.1)	512 (5.2)	0.003
No medical history	904 (13.6)	442 (12.5)	462 (14.8)	0.008
Dispatcher priority – n (%) (15,18)^b^				< 0.001
Priority 1	3249 (49.1)	1647 (46.9)	1602 (51.5)	
Priority 2	3071 (46.4)	1700 (48.4)	1371 (44.1)	
Priority 3	299 (4.5)	163 (4.6)	136 (4.4)	
Dispatch medical index^c^ – n (%) (12,19)^b^				
Chest pain/cardiac disease	991 (15.0)	514 (14.6)	477 (15.3)	0.427
Extremity/wound minor trauma	804 (12.1)	448 (12.8)	356 (11.5)	0.113
Uncertain information/suspicion of severe illness	726 (11.0)	341 (9.7)	385 (12.4)	0.001
Respiratory difficulties	708 (10.7)	412 (11.7)	296 (9.5)	0.004
Abdominal/urinary tract symptoms	704 (10.6)	306 (11.3)	308 (9.9)	0.079
EMS contact on multiple occasions^d^ – n (%)				0.580
One occasion	5450 (91.0)	2873 (90.8)	2577 (91.3)	
Two occasions	422 (7.0)	230 (7.3)	192 (6.7)	
Three occasions	75 (1.3)	39 (1.2)	36 (1.3)	
Four or more occasions	40 (0.7)	21 (0.7)	19 (0.7)	
Initial assessment of level of care – n (%)				
Hospital	5340 (80.3)	2763 (78.4)	2577 (82.4)	< 0.001
Emergency department	4920 (92.1)	2526 (91.4)	2394 (92.9)	0.045
Bypass emergency department^e^	420 (7.9)	237 (8.6)	183 (7.1)	
Referral to primary care	143 (2.1)	82 (2.3)	61 (2.0)	0.310
Stay on scene with increased social/home care	100 (1.5)	56 (1.6)	44 (1.4)	0.614
Stay on scene with advice on self-care/medication	1069 (16.1)	624 (17.7)	445 (14.2)	< 0.001
Mode of transport for patients initially assessed to hospital – n (%)				0.020
Ambulance	4979 (93.2)	2555 (92.5)	2424 (94.1)	
Patient transport	141 (2.6)	77 (2.8)	64 (2.5)	
Seated transport	159 (3.0)	94 (3.4)	65 (2.5)	
Police	20 (0.4)	17 (0.6)	3 (0.1)	
Own transportation	41 (0.8)	20 (0.7)	21 (0.8)	

^1^*P* values calculated for female, male groups

^a^The five most common medical history ICD-10 chapters, a patient can have more than one diagnosis

^b^Missing in each group respectively

^c^The five most common dispatch medical indices

^d^Calculated on indviduals respectively and number of EMS contacts in study period including renewed contact (total *n* = 6712)

^e^Bypass including pathway stroke, hip fracture, cardiac ICU, cath-lab, admission by EMS directly to geriatric ward, infection ward and psychiatric ED

**Table 2:** Patients initially assessed to hospital and non-transport patients

	Initial assessment to hospital	Non-transport	*P*
	*n*=5340	*n* = 1312	
Age – years (25th, 75th percentile)			
Median	68 (44,83)	59 (34,78)	< 0.001
Sex – n (%)			< 0.001
Female	2763 (51.7)	762 (58.1)	
Dispatcher priority – n (%) (26,7)^a^			< 0.001
Priority 1	2659 (50.0)	590 (45.2)	
Priority 2	2444 (46.0)	627 (48.0)	
Priority 3	211 (4.0)	88 (6.7)	
Dispatch medical index^b^ – n (%) (31,0)^a^			
Chest pain/cardiac disease	747 (14.1)	244 (18.7)	< 0.001
Extremity/ wound/minor trauma	699 (13.2)	105 (8.1)	< 0.001
Uncertain information/suspicion of severe illness	574 (10.8)	152 (11.6)	0.401
Respiratory difficulties	554 (10.4)	154 (11.7)	0.162
Abdominal/urinary tract symptoms	618 (11.6)	86 (6.6)	< 0.001
Time of day – n (%)			< 0.001
08:00–16:00	2431 (45.5)	475 (36.2)	
16:00–24:00	1902 (35.6)	536 (40.9)	
24:00–08:00	1007 (18.9)	301 (22.9)	
Time on scene – mins (25th, 75th percentile)			
Median	22 (14,30)	27 (19,37)	< 0.001
Medical history^c^ – n (%)			
Diseases of the circulatory system I00-I99	5250 (29.4)	887 (24.8)	< 0.001
Mental and behavioural disorders F00-F99	3024 (16.9)	787 (20.7)	< 0.001
Endocrine, nutritional and metabolic diseases E00-E89	1583 (8.9)	323 (9.0)	0.750
Diseases of the musculoskeletal system and connective tissue M00-M99	1212 (6.8)	237 (6.8)	0.768
Diseases of the digestive system K00-K95	1055 (5.9)	170 (4.8)	0.007
No medical history	658 (12.3)	246 (18.8)	< 0.001
Initial vital signs – median (25th, 75th percentile) (% deviating)^d^			
Respiratory rate/min (343,241)^a^	18 (16,20) (11.4)	16 (16,18) (1.6)	< 0.001
Saturation % (282,210)	97 (95,99) (5.4)	98 (96,99) (0.9)	< 0.001
Pulse rate/min (264,209)	86 (75,101) (8.3)	83 (74,93) (1.6)	< 0.001
Systolic blood pressure – mm/hg (369,240)	135 (120,150) (2.1)	130 (120,150) (0.3)	0.008
Diastolic blood pressure – mm/hg (947,333)	80 (70,90) (0.1)	80 (70,85) (0.1)	< 0.001
Temperature°C (762,332)	36.9 (36.5,37.3) (0.6)	36.8 (36.5,37.1) (0.1)	0.019
Level of consciousness – n (%) (20,37)			<0.001
RLS 1/ GCS 15^e^	4901 (92.1)	1224 (96.0)	
RLS 2,3/ GCS 13–10	310 (5.9)	43 (3.4)	
RLS ≥ 4/ GCS ≤ 8	109 (2.0)	8 (0.6)	
Prehospital triage level according to RETTS-A – n (%) (172,379)^a^			<0.001
Red	597 (11.6)	7 (0.8)	
Orange	1854 (35.9)	54 (5.8)	
Yellow	2254 (43.6)	341 (36.5)	
Green	463 (9.0)	531 (56.9)	
Prehospital field assessment according to RETTS-A^f^ – n (%) (161,377)^a^			
Chest thoracic pain	512 (9.9)	111 (11.9)	0.069
Abdominal/flank pain	535 (10.3)	59 (6.3)	<0.001
Respiratory distress/dyspnoa/breathing difficulties	454 (8.8)	91 (9.7)	0.350
Unspecific condition	349 (6.7)	142 (15.2)	<0.001
Injury/head trauma	324 (6.3)	28 (3.0)	<0.001
Under the influence of substances (alcohol, drugs) – n (%)	534 (10.0)	135 (10.3)	0.759
Prehospital medication – n (%)			
Any medication	1825 (34.2)	86 (6.6)	<0.001
Intravenous medication	1015 (19.0)	18 (1.4)	<0.001
All-cause mortality – n (%)			
≤ 7 days	111 (2.1)	16 (1.2)	0.042
≤ 30 days	243 (4.6)	31 (2.4)	<0.001
≤ 365 days	804 (15.1)	122 (9.3)	<0.001

^a^Missing in each group respectively

^b^The five most common dispatch medical indices

^c^The five most common medical history ICD-10 chapters, a patient can have more than one diagnosis

^d^Deviating vital signs: respiratory rate/min > 25 or < 8, oxygen saturation < 90%, pulse rate/min > 120 or < 40, systolic blood pressure < 90 mm/hg, diastolic blood pressure > 140 mm/hg, body temperature Celsius > 41 or < 35

^e^*RLS* Reaction level scale 1–8, *GCS* Glasgow coma scale 15–3

^f^The five most common EMS field assessments

### The EMS system in Sweden

Sweden is divided into 21 regions responsible for the health care within the region. The health care provided is tax funded, including prehospital care. EMS organisations use national/regional guidelines. It is mandatory according to legislation in Sweden that each ambulance is staffed by a registered nurse. Ambulance crew set-ups can be two nurses or one nurse and one EMT. The EMS nurse assesses the patient at the scene, have approximately 40 different types of drugs at her disposal, and performs treatments with the aid of guidelines. The EMS nurse has been given the responsibility to decide upon the level of care which includes 1. Assessed as requiring hospital (bypassing the ED for certain patient groups, for example in patients with suspected hip-fractures the EMS nurse writes a referral for x-ray and transports the patient directly to x-ray, 2. Arrange an appointment at primary care and 3. Treat and release and/or give advice on self-care. The EMS nurse also has the possibility to either contact home care and handover the patient if not assessed as an emergency or to contact social care if support is needed. To aid in the EMS nurse assessment at the scene, the Triage system RETTS-A is used.

### Triage system

The RETTS-A is a five-level triage system currently in use in the majority of the EDs and EMS organisations in Sweden. It was initially developed at the ED at Sahlgrenska University Hospital and is currently developed, maintained, and licensed by a Swedish company Predicare AB. The RETTS-A is similar to the Manchester Triage System (MTS) with emergency signs and symptoms (ESS). In order to identify patients at risk of deterioration at an early stage, vital signs (VS) have been added to all flow charts in RETTS-A (respiratory rate/min, oxygen saturation, pulse rate/min, blood pressure mm/hg, body temperature °C and level of consciousness). The ESS codes contain 58 charts with the most common presentations in the ED. The levels of severity both in ESS and VS are divided into the colours Red, Orange, Yellow, Green and Blue (not used in the EMS). Triage level Red is considered life-threatening, Orange potentially life-threatening, while Yellow and Green can wait in the ED without medical risk. Yellow is considered to be more urgent than Green. The highest colour of either the ESS or the VS becomes the final triage level. For example: a patient with chest pain and normal VS would be triaged to Red level according to the ESS if there is an ST-elevation on electrocardiogram (ECG) or if there is current chest pain and an affected general condition such as paleness or cold sweats. The EMS triage level follows the patient in the ED where handover to the ED nurse takes place and the triage level is reported. The patient will be reassessed in the ED at time-intervals depending on triage level. Patients triaged to Red level are always notified upon arrival to the ED and are assessed by a physician immediately.

### Time-critical diagnosis, deviating VS, and occurrence of complications

We defined a time-critical diagnosis as a condition, for example, myocardial infarction, stroke, or sepsis, which would initially require rapid management and a transfer to definitive care. Deviating VS was defined according to reference values set by the RETTS triage level Red or Orange (Additional file 1: Table S1) We also defined complications as one of the following conditions if they occurred within 48 h of the initial patient assessment by the EMS nurse: death, cardiac arrest, ventricular arrhythmias, status epilepticus, severe heart failure, hypotension, syncope, and unconsciousness or a deviation in VS according to RETTS-A red level. All cases were reviewed in hospital records up to 48 h from the initial EMS patient assessment.

### Statistical analysis

The results are presented as number (percentage) or median, except for days of inpatient care, where the mean has also been calculated. For two-group comparisons, as shown in Tables 1, 2, 3, and 5, the Mann-Whitney U test was used, while the Fisher’s exact test was used for continuous/ordered and dichotomous/categorical variables respectively. As shown in Table 4, age groups were divided into four quartiles based on the median age. To test for any association with age, Spearman’s rank statistics were used for continuous/ordered variables and the Kruskal-Wallis test was used for dichotomous/categorical variables. A two-group comparison was performed between the first assessment in the renewed ED 72-h group and the initial assessment in the group which was directly sent to hospital (Table 3). All the tests are two-sided and, because of the number of statistical tests performed in the study, *p*-values < 0.01 were considered to be significant. SPSS version 22 (IBM Corp, Armonk, NY, USA) was used for statistical analysis.

**Table 3:** Patients non-transported with 72 h ED attendance and patients initially assessed to hospital at first EMS contact

	ED attendance within 72 h	Initially assessed to hospital	*P*
	*n* = 126	*n* = 5340	
Age – median year (25th,75th percentile)	64 (36,82)	68 (44,83)	0.247
Sex – n (%)			0.787
Female	67 (53.2)	2763 (51.7)	
Mode of transport – n (%)			< 0.001
Ambulance	55 (44.4)	4979 (93.2)	
By own transportation	57 (45.2)	41 (0.8)	
Patient transport	12 (9.5)	141 (2.6)	
Seated transport	2(1.6)	159 (3.0)	
Police	0 (0.0)	20 (0.4)	
Dispatcher priority - n (%) (66,26)^a^			< 0.001
Priority 1	18 (30.0)	2659 (50.0)	
Priority 2	37 (61.7)	2444 (46.0)	
Priority 3	5 (8.3)	211 (4.0)	
Medical history – n (%)^b^			
Diseases of the circulatory system I00-I99	110 (27.3)	5250 (29.3)	0.376
Mental and behavioural disorders F00-F99	92 (22.8)	3024 (16.9)	0.002
Diseases of the musculoskeletal system and connective tissue M00-M99	37 (9.2)	1212 (6.8)	0.067
Endocrine, nutritional and metabolic diseases E00-E89	28 (6.9)	1583 (8.8)	0.216
Diseases of the digestive system K00-K95	25 (6.2)	1055 (5.9)	0.761
No medical history	19 (15.1)	658 (12.3)	0.339
Prehospital field assessent according to RETTS-A^c^ – n (%)^d^	1st assessment / 2nd assessment		
Unspecific condition, malaise	20 (20.2) / 12 (20.1)	349 (6.7)	< 0.001
Respiratory distress/dyspnoa/breathing difficulties	13 (14.9) / 8 (13.4)	454 (8.8)	0.055
Abdominal/flank pain	9 (10.3) / 8 (13.4)	535 (10.3)	1.000
Chest thoracic pain	5 (5.7) / 1 (1.7)	512 (9.9)	0.273
Nausea/vomiting	4 (3.2) / 0 (0.0)	43 (0.8)	0.022
Missing field assessment	39 (31.0) / 2 (3.3)	161 (3.0)	< 0.001
Prehospital triage level according to RETTS-A – n (%) (39 / 2, 172)^e^	1st triage / 2nd triage		< 0.001
Red	2 (2.3) / 6 (10.0)	597 (11.6)	
Orange	3 (3.4) / 15 (25.0)	1854 (35.9)	
Yellow	33 (37.9) / 31 (51.7)	2254 (43.6)	
Green	49 (56.3) / 6 (10.0)	463 (9.0)	
Management ED – n (%) (0, 14)^f^			
Admitted to in-patient care	58 (46.0)	2631 (49.4)	0.472
Extended examination/intervention^g^	22 (17.5)	774 (14.5)	0.371
Lab, drug administration, prescription	32 (25.4)	1412 (26.5)	0.839
Clinical examination/observation only	7 (5.6)	275 (5.2)	0.837
Patient managed by ED nurse, referral to primary care	3 (2.4)	54 (1.0)	0.144
Patient leaves without being seen or against medical advice	4 (3.2)	180 (3.4)	1.000
Days of in-patient care – n			
Mean (SD)	8.7 (8.8)	8.1 (9.5)	0.641
Median (25th,75th percentile)	5.5 (2.8,12)	5 (2,11)	0.633
Final hospital assessment, ICD-10^h^ – n (%) (11, 344)^f^			
Symptoms, signs and abnormal clinical and laboratory findings R00-R99	24 (20.9)	971 (19.4)	0.721
Injury, poisoning and certain other consequnces of external causes S00-S99, T00-T98	15 (13.0)	928 (18.6)	0.145
Mental and behavioural disorders F00-F99	14 (12.2)	423 (8.5)	0.174
Diseases of the circulatory system I00-I99	12 (10.4)	635 (12.7)	0.570
Disesases of the respiratory system J00-J99	7 (6.1)	418 (8.4)	0.494
Time-critical diagnosis (11,344)^f^	12 (10.4)	551 (11.0)	1.000
Deranged clinical signs/occurrence of complications within 48 h^i^	7 (5.6)	537 (10.1)	0.099
All-cause mortality - n (%)			
≤ 7 days	2 (1.8)	111 (2.1)	0.702
≤ 30 days	5 (4.0)	243 (4.6)	0.756
≤ 365 days	20 (15.9)	804 (15.1)	0.800

^a^Secondary dispatch priority for ED attendance within 72 h, n = 66 missing for patients transported by other means and *n* = 26 assignments missing dispatch priority

^b^The five most common medical history ICD-10 chapters, a patient can have more than one diagnosis

^c^The five most common EMS field assessments

^d^P values calculated on first assessment of ED attendance within 72 h group and initially assessed to hospital

^e^Missing triage level for first assessment, second assement and patients initially assessed to hospital

^f^Missing in each group respectively

^g^X-ray, computed tomography, ultrasound, magnetic resonance, lumbar puncture, suturing, proctoscopy

^h^The five most common final hospital ICD-10 chapters

^i^Occurrence of life-threatening events in ambulance, ED or ward within 48 h

**Table 4:** Patient distribution of age in quartiles based on median age

	Quartiles				
	1st	2nd	3rd	4th	*P*
	16–42	43–65	66–82	83–106^1^	
	*n* = 1705	*n* = 1552	*n* = 1812	*n* = 1583	
Sex – n (%)					
Female	908 (53.3)	721 (46.5)	892 (49.2)	1004 (63.4)	<0.001
Dispatcher priority – n (%) (14,6,10,3)^a^					<0.001
Priority 1	965 (57.1)	832 (53.8)	862 (47.8)	590 (37.3)	
Priority 2	674 (39.9)	660 (42.7)	851 (47.2)	886 (56.1)	
Priority 3	52 (3.1)	54 (3.5)	89 (4.9)	104 (6.6)	
Dispatch medical index^b^ – n (%) (13,7,10,1)^a^					
Chest pain/cardiac disease	158 (9.3)	269 (17.4)	322 (17.9)	242 (15.3)	<0.001
Extremity wound/minor trauma	120 (7.1)	130 (8.4)	239 (13.3)	315 (19.9)	<0.001
Uncertain information/suspicion of severe illness	158 (9.3)	166 (10.7)	215 (11.9)	187 (11.8)	0.056
Respiratory difficulties	100 (5.9)	118 (7.6)	255 (14.2)	235 (14.9)	<0.001
Abdominal/urinary tract symptoms	205 (12.1)	179 (11.6)	184 (10.2)	136 (8.6)	0.006
Medical history^c^ – n (%)					
Diseases of the circulatory system I00-I99	81 (2.5)	795 (18.7)	2415 (34.1)	2846 (41.5)	<0.001
Mental and behavioural disorders F00-F99	1486 (45.8)	1135 (26.7)	691 (9.8)	499 (7.3)	<0.001
Endocrine, nutritional and metabolic diseases E00-E89	163 (5.0)	397 (9.3)	795 (11.2)	551 (8.0)	< 0.001
Diseases of the musculoskeletal system and connective tissue M00-M99	84 (2.6)	251 (5.9)	535 (7.6)	579 (8.4)	< 0.001
Diseases of the digestive system K00-K95	186 (5.7)	304 (7.1)	413 (5.8)	322 (4.7)	< 0.001
No medical history	600 (35.2)	221 (14.2)	59 (3.3)	24 (1.5)	< 0.001
Time of day – n (%)					<0.001
08:00–16:00	548 (32.1)	621 (40.0)	877 (48.4)	860 (54.3)	
16:00–24:00	724 (42.5)	598 (24.5)	643 (35.5)	473 (29.9)	
24:00–08:00	433 (25.4)	333 (21.5)	292 (16.1)	250 (15.8)	
Time on scene – mins (25th, 75th percentile)					
Median	19 (12,28)	20 (14,28)	24 (17,33)	27 (19,35)	<0.001
Initial vital signs – median (25th, 75th percentile) (% deviating)^d^					
Respiratory rate/min (198,154,133,99)^a^	18 (16,20) (4.8)	18 (16,20) (6.4)	18 (16,20) (12.5)	18 (16,22) (14.4)	< 0.001
Saturation % (167,130,108,87)	99 (97,100) (1.2)	98 (96,99) (2.8)	97 (95,98) (6.8)	96 (94,98) (7.2)	< 0.001
Pulse rate/min (160,126,107,80)	90 (78,104) (8.5)	86 (75,100) (7.3)	85 (74,100) (7.3)	83 (71,97) (5.5)	< 0.001
Systolic blood pressure – mm/hg (219,164,132,94)	120 (112,135) (1.1)	140 (120,150) (1.8)	140 (120,160) (2.1)	140 (120,160) (1.9)	<0.001
Diastolic blood pressure – mm/hg (430,316,291,243)	80(70,85) (0.0)	80(75,90) (0.1)	80 (70,90) (0.3)	80 (70,90) (0.0)	0.187
Temperature °C (411,303,230,150)	36.9 (36.5,37.3) (0.5)	36.8 (36.4,37.1) (0.6)	36.9 (36.5,37.3) (0.6)	36.9 (36.5,37.3) (0.4)	0.164
Level of consciousness – n (%) (11,18,16,12)					<0.001
RLS 1/ GCS 15^e^	1521 (89.8)	1410 (91.9)	1699 (94.6)	1495 (95.2)	
RLS 2,3/ GCS 13–10	137 (8.1)	94 (6.1)	69 (3.8)	53 (3.3)	
RLS ≥4/ GCS ≤ 8	36 (2.1)	30 (2.0)	28 (1.6)	23 (1.5)	
Prehospital triage level according to RETTS-A – n (%) (192,144,107,108)^a^					0.525
Red	122 (8.1)	140 (9.9)	192 (11.3)	150 (10.2)	
Orange	507 (33.5)	470 (33.4)	507 (29.7)	424 (28.7)	
Yellow	621 (41.0)	561 (39.8)	738 (43.3)	675 (45.8)	
Green	263 (17.4)	237 (16.8)	268 (15.7)	226 (15.3)	
Prehospital field assessment according to RETTS-A^f^ – n (%) (192,143,104,99)^a^					
Chest/thoracic pain	108 (7.1)	177 (12.4)	202 (11.7)	136 (9.1)	<0.001
Abdominal/flank pain	194 (12.7)	174 (12.3)	134 (7.8)	92 (6.2)	<0.001
Respiratory distress/dyspnoa/breathing difficulties	42 (2.8)	79 (5.6)	228 (13.3)	196 (13.2)	<0.001
Unspecific condition	49 (3.2)	84 (6.0)	164 (9.6)	194 (13.1)	<0.001
Injury/head trauma	99 (6.5)	79 (5.6)	86 (5.0)	88 (5.9)	0.320
Under the influence of substances (alcohol, drugs) – n (%)	341 (20.0)	220 (14.2)	93 (5.1)	15 (0.9)	<0.001
Prehospital medication – n (%)					
Any medication	424 (24.9)	439 (28.3)	573 (31.6)	475 (30.0)	<0.001
Intravenous medication	237 (13.9)	245 (15.8)	289 (15.9)	262 (16.6)	0.170
Level of care – n (%)					
Hospital	1274 (74.7)	1233 (79.4)	1486 (82.0)	1347 (85.1)	<0.001
Emergency department	1174 (92.2)	1162 (94.2)	1380 (92.9)	1204 (89.4)	<0.001
Bypass emergency department^g^	100 (7.8)	71 (5.8)	106 (7.1)	143 (10.6)	
Referral to primary care	46 (2.7)	28 (1.8)	46 (2.5)	23 (1.5)	0.041
Stay on scene with initiated/increased social/home care	12 (0.7)	15 (1.0)	32 (1.8)	40 (2.6)	<0.001
Stay on scene with advice on self-care/medication	373 (21.9)	276 (17.8)	248 (13.7)	17 2(10.9)	<0.001
ED admission within 72 h	38 (2.9)	26 (2.1)	32 (2.1)	30 (2.2)	0.437
Mode of transport – n (%)					0.852
Ambulance	1190 (93.4)	1148 (93.1)	1391 (93.6)	1250 (92.8)	
Patient transport	10 (0.8)	21 (1.7)	49 (3.3)	61 (4.5)	
Seated transport	47 (3.7)	42 (3.4)	38 (2.6)	32 (2.4)	
Police	13 (1.0)	7 (0.6)	0 (0.0)	0 (0.0)	
Own transportation	14 (1.1)	15 (1.2)	8 (0.5)	4 (0.3)	
Management ED – n (%) (3, 1,7,3)^a^					
Admitted to in-patient care	346 (27.2)	508 (41.2)	878 (59.4)	899 (66.9)	< 0.001
Extended examination/intervention^h^	262 (20.6)	198 (16.1)	171 (11.6)	143 (10.6)	< 0.001
Lab, drug administration, prescription	430 (33.8)	370 (30.0)	347 (23.5)	265 (19.7)	< 0.001
Clinical exam/observation only	124 (9.8)	76 (6.2)	48 (3.2)	27 (2.0)	< 0.001
Patient managed by ED nurse, referral to primary care	23 (1.8)	16 (1.3)	12 (0.8)	3 (0.2)	< 0.001
Patient leaves without being seen or against medical advice	86 (6.8)	64 (5.2)	23 (1.6)	7 (0.5)	< 0.001
Days of in-patient care – n					
Mean (SD)	5.7 (12.0)	6.5 (10.1)	8.8 (9.6)	9.2 (7.3)	< 0.001
Median (25th,75th percentile)	2 (1,5)	4 (2,7)	6 (3,11)	7 (4,13)	< 0.001
Final hospital assessment, ICD-10^i^ – n (%) (140,104,62,38)^a^					
Symptoms, signs and abnormal clinical and laboratory findings R00-R99	237 (20.9)	241 (21.3)	269 (18.9)	224 (17.1)	0.030
Injury, poisoning and certain other consequences of external causes S00-S99, T00-T98	261 (23.0)	204 (18.1)	215 (15.1)	248 (18.9)	<0.001
Mental and behavioural disorders F00-F99	215 (19.0)	130 (11.5)	50 (3.5)	28 (2.1)	<0.001
Diseases of the circulatory system I00-I99	22 (1.9)	123 (10.9)	250 (17.6)	240 (18.3)	<0.001
Disesases of the respiratory system J00-J99	39 (3.4)	62 (5.5)	174 (12.2)	143 (10.9)	<0.001
Time-critical diagnosis (140,104,62,38)^a^	109 (9.6)	95 (8.4)	185 (13.0)	162 (12.4)	<0.001
Deranged clinical signs/occurrence of complications within 48 h^j^	85 (6.7)	106 (8.6)	188 (12.7)	158 (11.7)	<0.001
All-cause mortality – n (%)					
≤ 7 days	6 (0.4)	13 (0.8)	44 (2.4)	64 (4.0)	<0.001
≤ 30 days	6 (0.4)	27 (1.7)	86 (4.7)	155 (9.8)	<0.001
≤ 365 days	21 (1.2)	85 (5.5)	319 (17.6)	501 (31.6)	<0.001

^a^Missing in each group respectively

^b^The five most common dispatch medical indices

^c^The five most common medical history ICD-10 chapters, a patient can have more than one diagnosis

^d^Deviating vital signs: respiratory rate/min > 25 or < 8, oxygen saturation < 90%, pulse rate/min > 120 or < 40, systolic blood pressure < 90 mm/hg, diastolic blood pressure > 140 mm/hg, body temperature Celsius > 41 or < 35

^e^*RLS* Reaction level scale 1–8, *GCS* Glasgow coma scale 15–3

^f^The five most common EMS field assessments

^g^Bypass including pathway stroke, hip fracture, cardiac ICU, cath-lab, admission by EMS directly to geriatric ward, infection ward and psychiatric ED

^h^X-ray, computed tomography, ultrasound, magnetic resonance, lumbar puncture, suturing, proctoscopy

^i^The five most common final hospital ICD-10 chapters

^j^Occurrence of life-threatening events in ambulance, ED or ward within 48 h

## Results

Of the total number of assignments, the median age was 66 years and 86.4% of the patients had a past medical history, with circulatory diagnoses such as hypertension, stroke, myocardial infarction and heart failure as the most common (28.6%). Psychiatric disorders such as anxiety, depression, and substance abuse were the next most common previously known diagnoses (17.8%). The dispatcher assigned life-threatening priority (1) in 49.1% of all the assignments, and males received higher priority. In total, 80.3% of the patients were assessed as requiring hospital care by the EMS nurse and were transported by the ambulance at the scene (93.2%). Males were assessed as requiring hospital care more frequently (Table 1).

### Initially assessed as requiring hospital care and non-transported

A total of 1312 patients (19.7%) were initially non-transported. The median age was higher for the patients assessed as requiring hospital care compared to non-transported patients. Cases given priority 1 by the dispatcher were more commonly assessed by the EMS nurse as requiring hospital care. The most common DMI, ‘chest pain/cardiac disease’, was more common in the non-transported group (18.7%). On the other hand, the DMIs ‘extremity/wound/trauma’ and ‘abdominal/urinary tract’ were more common in patients initially assessed as requiring hospital care. There was a higher percentage of non-transported patients in the evening and during the night. If the patient had a past medical history of a circulatory diagnosis, including risk factors such as prior stroke, myocardial infarction, or hypertension, the patient was more likely to be assessed as requiring hospital care. Of the patients who were non-transported, ‘mental and behavioural disorders’ or no medical history were more common. Triage level Green was more frequently associated with non-transport. ‘Chest/thoracic pain’ was the most common EMS nurse-assessed condition, with no difference between groups. Assessment with ‘abdominal/flank pain’ and ‘injury/head trauma’ were more common among patients who were transported to hospital, while ‘unspecific condition’ was more common among non-transported patients. Only 34% of the patients who received an ALS ambulance and were assessed by the EMS nurse as requiring hospital care received any medication; 19% received intravenous medication (Table 2).

### Patients non-transported with ED admission within 72 h

A total of 126 (9.6%) patients were admitted to the ED within 72 h (ED72) with a condition related to the initial assessment by the EMS nurse. Transport by ambulance was lower in the ED72 group (44.4%) compared with the initially assessed as requiring hospital care (IAH) group (93.2%). The dispatch priority was lower in the ED72 group than in the IAH group. In the ED72 group, it was more common to have a medical history of ‘mental and behavioural disorders’ than in the IAH group. The EMS nurse assessed 20% of the patients as ‘unspecific condition, malaise’ in the first and second assessment in the ED72 group, which was higher than in the IAH group. There was a higher percentage of missing triage assessments according to RETTS-A in the ED72 group than in the IAH group, but the missing triage assessment decreased in the second assessment from 31.0 to 3.3%. More patients were assessed as requiring a higher triage level in the ED72 group in the second assessment than in the first assessment, with 35% found in the Red and Orange categories in the second assessment, compared with 5.7% in the first assessment. In the ED72 group, 46% were admitted to inpatient care, with a median stay of 5.5 days, and another 17.5% of the patients received ‘extended examination/intervention’ in the ED. There was no difference between ED72 and IAH when comparing admission to inpatient care, ED management, or days of inpatient care. The most common ICD diagnoses in the ED72 group were found in the ‘Symptoms, signs and abnormal clinical findings (R00-R99)’, for example dyspnoea and chest pain. Of the ED72 patients, a total of 12 patients (10.4%) were diagnosed with a time-critical condition, and seven patients (5.6%) had an adverse event within the first 48 h (Table 3).

### Age distribution in quartiles and patient assessment

Most of the patients were found in the third quartile (Q3), aged 66–82 years. The younger patients (Q1) received priority 1 to a greater extent by dispatch (57.1%), compared with the oldest patients (Q4) (37.3%). A DMI of ‘chest/thoracic’ pain was more common in Q2 and Q3. A DMI of ‘extremity/wound/minor’ trauma was more commonly in the elderly (Q4) and ‘abdominal/urinary tract’ was more common in the younger patients (Q1 and Q2). In Q4, 41.5% had a history of circulatory diseases. By contrast, among patients in Q1, ‘mental and behavioural disorders’ were more common (45.8%). The EMS was dispatched to patients in Q1 to a greater extent in the evening (42.5%) and during the night (25.4%) compared with patients in the other quartiles, whereas the EMS was dispatched more often to patients in Q4 during office hours (54.3%). There was a trend towards a more frequent initial hospital assessment for older patients, with 85.1% of the patients in Q4 transported to hospital compared with 74.7% of the patients in Q1. Less time was spent at the scene for the younger patients, ranging from 19 min (median) in Q1 to 27 min (median) in Q4. Vital Signs deviated more frequently from normal among the elderly (Q4), with the exception of pulse rate and degree of consciousness, where a reverse trend was observed. Substance abuse at the time of assessment decreased markedly with increasing age. The elderly were assessed by the EMS nurse at the scene as ‘unspecific condition’ more frequently than younger patients. Assessed conditions such as abdominal pain was more common at younger ages. In hospital, the majority of the patients in Q3 and Q4 were admitted to inpatient care, with a median length of stay of six and 7 days, compared with Q1 where only 27% of the patients were admitted to inpatient care, with a median stay of 2 days. In Q1, 18.4% were discharged from the ED with no intervention, were referred to PC, or left the ED without being seen by a physician. A psychiatric diagnosis at hospital discharge was also more common in Q1 (19.0%). Patients in Q4 received a diagnosis relating to the circulatory system to a greater extent (18.3%). Older patients also received prehospital medication more often compared with younger patients (Table 4).

### All-cause mortality

Among all the patients, 127 (1.9%) died within 7 days, 274 (4.1%) died within 30 days and, 1 year after the EMS visit, a total of 926 (13.9%) patients had died. All-cause mortality for 30 days was significantly higher in transported compared to non-transported patients (4.6% vs 2.4%) as well as one-year mortality (15.1% vs 9.3%). Albeit, no significant difference was found between the two groups for seven-day mortality (2.1% vs 1.2%) (Table 2). There were also no significant differences found between ED72 and IAH group for seven-day mortality (1.8% vs 2.1%), 30-day mortality (4.0% vs 4.6%) and one-year mortality (15.9% vs 15.1%) (Table 3). Most of the deaths were found in the oldest age group (Q4), with a seven-day mortality of 4.0% and 30-day mortality of 9.8% (Table 4). Patients with limited triage had the highest risk of death within 7 days (3.6%) compared with patients with full triage (1.4%) (Table 5).

**Table 5:** Patients assessed with full triage or limited triage

	Full triage	Limited triage	*P*
	*n* = 5150	*n* = 1502	
Age – years (25th, 75th percentile)			
Median	69 (45,83)	57 (34,76)	<0.001
Sex – n (%)			
Female	2758 (53.6)	767 (51.1)	0.089
Dispatcher priority – n (%) (16,17)^a^			<0.001
Priority 1	2436 (47.4)	813 (54.7)	
Priority 2	2485 (48.4)	586 (39.5)	
Priority 3	213 (4.1)	86 (5.8)	
Dispatch medical index^b^ – n (%) (19,12)^a^			
Chest pain/cardiac disease	834 (16.3)	157 (10.5)	<0.001
Extremity/wound/minor trauma	612 (12.9)	192 (11.9)	0.322
Uncertain information/suspicion of severe illness	570 (11.1)	156 (10.5)	0.510
Respiratory difficulties	602 (11.7)	106 (7.1)	<0.001
Abdominal/urinary tract symptoms	624 (12.2)	80 (5.4)	<0.001
Medical history^c^ – n (%)			
Diseases of the circulatory system I00-I99	5135 (30.0)	1002 (23.1)	<0.001
Mental and behavioural disorders F00-F99	2616 (15.3)	1195 (27.5)	<0.001
Endocrine, nutritional and metabolic diseases E00-E89	1558 (9.1)	348 (8.0)	0.024
Diseases of the musculoskeletal system and connective tissue M00-M99	1190 (7.0)	259 (6.0)	0.020
Diseases of the digestive system K00-K95	1046 (6.1)	179 (4.1)	<0.001
No medical history	634 (12.3)	270 (18.0)	<0.001
Time of day – n (%)			0.037
08:00–16:00	2294 (44.5)	612 (40.7)	
16:00–24:00	1848 (35.9)	590 (39.3)	
24:00–08:00	1008 (19.6)	300 (20.0)	
Time on scene – mins (25th, 75th percentile)			
Median	23 (16,31)	21 (13,30)	<0.001
Prehospital triage level – n (%) (0, 551)^a^			0.655
Red	489 (9.5)	115 (12.1)	
Orange	1609 (31.2)	299 (31.4)	
Yellow	2244 (43.6)	351 (36.9)	
Green	808 (15.7)	186 (19.6)	
Prehospital field assessment according to RETTS-A^d^ – n (%) (0,538)^a^			
Chest/thoracic pain	537 (10.4)	86 (8.9)	0.164
Abdominal/flank pain	548 (10.6)	46 (4.8)	<0.001
Respiratory distress/dyspnoa/breathing difficulties	504 (9.8)	41 (4.3)	<0.001
Unspecific condition	423 (8.2)	68 (7.1)	0.245
Injury head trauma	266 (5.2)	86 (8.9)	<0.001
Under the influence of substances (alcohol, drugs) – n (%)	423 (8.2)	246 (16.4)	<0.001
Prehospital medication – n (%)			
Any medication	1581 (30.7)	330 (22.0)	<0.001
Intravenous medication	852 (16.5)	181 (12.1)	< 0.001
Level of care – n (%)			
Hospital	4394 (85.3)	946 (63.0)	<0.001
Emergency department	4072 (92.7)	848 (89.6)	0.002
Bypass emergency department^e^	322 (7.3)	99 (10.4)	
Referral to primary care	93 (1.8)	50 (3.3)	0.001
Stay on scene with increased social/home care	61 (1.2)	39 (2.6)	<0.001
Stay on scene with advice on self-care/medication	602 (11.7)	467 (31.1)	<0.001
ED admission within 72 h	76 (10.1)	50 (9.0)	0.570
Mode of transport to hospital – n (%)			0.001
Ambulance	4120 (93.8)	859 (90.8)	
By own transportation	32 (0.7)	9 (1.0)	
Patient transport	117 (2.7)	24 (2.5)	
Seated transport	121 (2.8)	38 (4.0)	
Police	16 (1.7)	4 (0.1)	
Management ED – n (%) (3,11)^a^			
Admitted to in-patient care	2208 (50.4)	423 (44.6)	0.002
Extended examination/intervention^f^	593 (13.5)	181 (19.2)	<0.001
Lab, drug administration, prescription	1203 (27.4)	209 (22.2)	0.001
Clinical examination/observation only	187 (4.3)	88 (9.3)	<0.001
Patient managed by ED nurse, referral to primary care	43 (1.0)	11 (1.2)	0.591
Patient leaves without being seen or against medical advice	149 (3.4)	31 (3.3)	0.921
Days of in-patient care – n			
Mean (SD)	8.0 (8.8)	8.8 (12.6)	0.097
Median (25th,75th percentile)	5 (3,11)	5 (2,11)	0.112
Final hospital assessment, ICD-10^7^ – n (%) (83,261)^a^			
Symptoms, signs and abnormal clinical and laboratory findings R00-R99	857 (20.7)	114 (13.2)	< 0.001
Injury, poisoning and certain other consequences of external causes S00-S99, T00-T98	693 (16.8)	235 (27.3)	< 0.001
Mental and behavioural disorders F00-F99	266 (6.4)	157 (18.2)	<0.001
Diseases of the circulatory system I00-I99	530 (12.8)	105 (12.2)	0.653
Diseases of the respiratory system J00-J99	383 (9.3)	35 (4.1)	<0.001
Time-critical diagnosis (83,261)^a^	416 (10.1)	135 (15.6)	< 0.001
Deranged clinical signs/ occurrence of complications within 48 h^h^	418 (9.5)	119 (12.6)	0.006
All-cause mortality – n (%)			
≤ 7 days	89 (1.7)	57 (3.8)	< 0.001
≤ 30 days	217 (4.2)	74 (4.9)	0.234
≤ 365 days	741 (14.4)	175 (11.7)	0.006

^a^Missing in each group respectively

^b^The five most common dispatch medical indices

^c^The five most common medical history ICD-10 chapters, a patient can have more than one diagnosis

^d^The five most common EMS field assessments

^e^ypass including pathway stroke, hip fracture, cardiac ICU, cath-lab, admission by EMS directly to geriatric ward, infection ward and psychiatric ED

^f^X-ray, computed tomography, ultrasound, magnetic resonance, lumbar puncture, suturing, proctoscopy

^g^The five most common final hospital ICD-10 chapters

^h^Occurrence of life-threatening events in ambulance, ED or ward within 48 h

### Adherence to RETTS triage system

In all, 22.6% of patients were not triaged with an ESS assessment + VS, as the guidelines prescribe. Patients with limited triage were younger and were often assessed as priority 1 by dispatch. Patients with full triage were mostly assigned with a DMI of ‘chest pain/cardiac disease’, ‘abdominal/urinary tract symptoms’, or ‘respiratory difficulties’. A history of a circulatory disease was more common in the full triage group, whereas a psychiatric disorder was the most common medical history in the limited triage group. When the EMS nurse assessed the patient at the scene, the field assessments of ‘abdominal/flank pain’ and ‘respiratory distress/breathing difficulties’ were associated with full triage, whereas limited triage was associated with ‘injury/head trauma’. Being under the influence of substances at the time of the EMS nurse’s assessment was also associated with limited triage. Being assessed as requiring other levels of care was also associated with limited triage, as well as transport by means other than an ambulance and being given less medication. Among the patients who were initially assessed as requiring hospital care, patients were more commonly admitted to inpatient care if they received full triage, had laboratory tests taken, received medication in the ED, or were prescribed medication. Patients in the limited triage group more frequently had an ‘extended examination’ or ‘clinical examination/observation’. Furthermore, these patients more frequently had a diagnosis in the ICD chapters ‘Symptoms, signs and abnormal clinical and laboratory findings’, ‘Injury, poisoning and certain other consequences of external causes’, or a psychiatric diagnosis including intoxication at hospital discharge. Finally, these patients more frequently received a final diagnosis of a time-critical condition and had a higher frequency of deviating VS (RETTS-A Red) and complications within 48 h (Table 5).

## Discussion

In the present study, we aimed to describe patient characteristics and to evaluate patients in an unselected EMS population with regard to the initial EMS nurse assessment and level of care. To our knowledge, there have been few studies in a prehospital context containing a manual review of the patient process and the assessments/decisions performed at each step from the initial EMS call to hospital discharge/stayed at the scene. Field triage protocols in the EMS is not new but have been used in trauma patients for decision of transportation to the appropriate receiving hospital [17]. However, using a triage system at the scene for all patient conditions and basing transport decisions on triage level is a new development internationally. Moreover, the triage colour set by the EMS nurse at the scene is the same one that applies in the ED.

### Transported and non-transported patients

The median age of non-transported patients was almost 10 years younger than that of patients transported to hospital, indicating that younger patients are more frequently assessed by the EMS nurse with conditions not requiring hospital resources. However, over-triage exists at dispatch, with 45% of the non-transported patients assigned to priority 1. In a study from the UK, 41% of the patients were not transported to hospital, twice the number in this study, and specific factors for non-transport were age, sex, social deprivation, time of call, reason for call, urgency level, and competence of EMS crew [18], with findings similar to those in the current study. Of patients in our study transported to the ED, 36.1% were discharged with a referral to PC, had laboratory tests taken, or received a prescription or a clinical examination and could then be discharged. This indicates that more patients in this study could have been assessed as requiring a lower level of care than ED/hospital care. However, in the present situation, there are challenges for the EMS nurses making non-transport decisions. Despite not needing hospital resources, patients may need a physical examination and/or prescription. The limited accessibility of PC may lead to the majority of patients staying at the scene, with an interruption in the chain of care and no follow-up other than renewed contact with the EMS or ED. For instance, almost 12% of patients triaged with ‘chest pain’ remained at the scene, with more males assessed as requiring hospital care. The average age for non-transported patients with chest pain was 53 years and an ECG was obtained in 93 (83.8%) patients, and the majority of the patients were triaged to the lower levels Green and Yellow (94.6%). Non-transport decision in chest pain may be due to a number of reasons for example patients with psychiatric disorders and with psychosomatic symptoms, patients with known angina and symptoms in remission upon arrival or patients with gastrointestinal conditions such as gastritis or symptoms of musculoskeletal origin. Given the relatively low frequency of digitally transmitted ECG to the cardiac unit for consultation in this group (0.6%) (otherwise there is a low threshold for the EMS nurse to transmit ECG in chest pain patients with the aim of direct admission to cardiac unit) indicates that the EMS nurse had a very low suspicion of a cardiac related time-critical condition. However, previous studies have reported the under-recognition of heart disease among females, and the clinical presentation may differ. For example, females with chest pain are less frequently transported using lights and sirens, and are administered fewer drugs (i.e., aspirin) [19, 20]. Sex disparities in favour of male patients have also been reported in a study of EMS stroke recognition [21]. The introduction of point-of-care testing has been shown to be feasible in the EMS [22] and may aid further in complex assessments.

### ED admission within 72 h

Of the non-transported patients, 9.6% attended the ED, and 4.4% of the total non-transport group were hospitalised within 72 h. A systematic review reported 6.4–25.8% ED attendance and hospitalisation of 4.5–12.1% (72 h) among non-transported patients [23]. In our study, a past medical history of psychiatric disorders was higher in the ED72 group than in the IAH group, indicating the possibility of confirmation bias by the EMS nurse that may have led to a less appropriate decision on the first visit. Risk factors in the decision-making process resulting in confirmation bias or anchoring may contribute to an inappropriate clinical decision with the risk of an adverse event [24]. This is worrying, as the majority of the ED72 patients were initially assessed as having an ‘unspecific condition’, indicating difficulties in the assessment and patients presenting with vague symptoms. The large amount of uncertainty and lack of assessment is also a concern, considering that 10.4% of the patients in the ED72 group (0.9% of the total non-transported group) had a time-critical condition and were delayed. The majority of these patients had a stroke or sepsis. Similar findings have been reported from a Dutch study, in which 1.0% of non-transported patients with an urgent diagnosis required admission [25]. Previous studies have reported that 9–11% of non-transported patients are being under-triaged, at risk of clinical deterioration; thus, patient safety is jeopardised when paramedics or technicians make non-transport decisions for patients who need hospital emergency care resources [26–29]. Older age and abnormal VS are two predictors of adverse events in non-transported patients, and increased EMS crew competence has shown a reduction in ED transportation [30, 31]. Another study, which compared EMS nurses and physician assistants (PA) in assessing patients at the scene, reported that PA used a more medical diagnostic approach and assessed more patients to stay at the scene. The PA also consulted more medical specialists in the decision-making process [32]. However, it is not clear which type of additional training that is needed [23]. Non-transport decisions by EMS nurses are based on several factors, including experience, education, confidence, and guidelines [33]. If non-transported patients with a time-critical diagnosis within 72 h in our study are extrapolated to the whole EMS organisation in the study, then there are 150 non-transported patients with a time-critical condition every year. For example, older patients with a stroke presenting with vague symptoms are difficult to differentiate from those without stroke. In a previous Swedish study of patients with a hospital diagnosis of transient ischaemic attack/stroke, 2.6% of them had an interrupted transport due to lack of suspicion of the disease by the EMS nurse. The majority of them had vague symptoms of vertigo or disturbed balance, so instruments to aid in the assessment are called for [34]. A low threshold for physician referral at the ED, for physician-staffed mobile teams or for PC when applicable, is suggested in assessments of elderly patients in particular.

### Age distribution and EMS nurse assessment

Frail older patients with atypical presentations are common and have more adverse events, with a higher risk of hospitalisation, and these patients run an increased risk of being misdiagnosed [35]. For this reason, detecting frailty is essential in the first encounter with an older patient, and this may affect outcome. At the same time, many of the frail elderly may not benefit from being transported to the ED. Introducing specially allocated ambulances with geriatric and EMS competence, in close collaboration with home teams of geriatric physicians, could be an alternative to assess and care for this patient group. On the other hand, the majority of the patients with a medical history of psychiatric disorders were in contact during the evening and night, and they were less likely to be transported to the ED. Previous studies have reported that a greater number of younger non-urgent patients arrived at the ED by ambulance in the evenings [36, 37]. A more thorough assessment from dispatch with the support of enhanced decision systems may reduce the number of younger patients visited by the EMS, as many of them may be eligible for other care alternatives. However, suicidal behaviour in combination with substance abuse is not uncommon, and an at-the-scene EMS nurse assessment may be appropriate in many of these cases. A unit with one EMS nurse and one psychiatric specialist nurse has been introduced in the study organisation in the evenings to address this patient category to reduce transport to hospital and the allocation of ALS ambulances.

### All-cause mortality

In this study, the all-cause mortality of non-transported patients was 2.1% within 7 days. Other studies have reported rates between 0.3–0.7% for seven-day mortality [30, 38–40]. However, of the non-transported patients who died, decisions were made with relatives and primary care physicians on end-of-life care in several cases. Excluding these, only five patients were classified as inappropriately associated with death, giving 0.38% mortality within 7 days that could have been avoided if initially assessed as needing to be transported to hospital. On the other hand, adverse events have been associated with ED admission among elderly patients. The ED has thus been regarded as a high-risk environment, and patients over 65 are at greater risk of adverse events both at the ED and during in-patient care if they have a prolonged stay at the ED [41, 42]. Other options for elderly patients, including home visits by geriatric teams and pathways to inpatient care, might be feasible.

### Adherence to RETTS-A triage system

The RETTS-A triage system is mandatory when assessing patients in the EMS organisation in our study. However, RETTS-A was not created for non-transport decisions. In this study, 22.6% of all patients were found to have had limited triage with either some VS or ESS missing. The most common missing VS were diastolic blood pressure (19.2%) and body temperature (16.4%). Patients with time-critical conditions were more frequently associated with limited triage than full triage. An explanation for this may be that the EMS nurse may be occupied with an A or B problem and has insufficient time to obtain a temperature given that the patient is already triaged to highest level based on another VS or ESS. However, recording of all VS is essential for example: body temperature in a septic patient, pulse rate in detection of tachyarrhythmias, oxygen saturation in chronic heart failure or pulmonary emboli. Of the total patient population in this study relatively few had a deviating VS (RETTS-A Red or Orange level) and previous studies have reported that the risk of in-hospital death and adverse events are predicted by the number of VS deviating from normal (oxygen saturation, respiratory rate, systolic blood pressure, and level of consciousness) [43]. This indicates that a combination of VS with small deviations from normal may be of importance when it comes to the early identification of candidates for deterioration. In order to optimize the early evaluation a full set of VS is required for risk calculation.

However, another study in the ED reported that VS alone may not be conclusive in the detection of seriously ill elderly patients [44]. This suggests that 1) VS defined as normal in RETTS-A, i.e., systolic blood pressure of 110 mmHg, may represent a severely ill patient if he/she is older than 65 years [45], and 2) the chief complaint with risk stratification (ESS) may be of value in discriminating patients with normal VS. However, 35% of the patients in this study were assessed as having a potentially life-threatening condition (Orange) according to RETTS-A triage, and the majority of the patients received their final triage colour based on ESS alone, indicating potential over-triage. On the other hand, adherence to RETTS-A triage was greater for patients with ‘chest pain’, whereas patients with ‘injury/head trauma’ were more common among those with limited triage. Prior studies in the ED have shown adherence rates of 61–65% when utilising the MTS and Emergency Severity Index [46]. In the light of this, a full triage adherence rate of 77.4% for a triage system developed for the ED but utilised in the prehospital setting seems acceptable, with poorer adherence found in non-transported patients and the most critical cases.

On the other hand, poorer adherence to triage guidelines has been reported to yield greater under-triage for trauma patients [47]. This may be a threat to patient safety, especially for patients requiring early critical resources, and the Glasgow Coma Scale (GCS) has been reported to be the most important VS regarding intensive care unit (ICU) admission and death [48]. In this study, 92.1% of the patients were assessed with GCS 15, giving a low percentage of patients with a change in mental status. However, intoxicated patients with a change in mental status at the scene should be carefully assessed, and any potential time-critical condition should be ruled out. Of the non-transported patients, 28.9% were missing a triage level. This suggests that non-transported patients are frequently assessed without full triage. Given that elderly patients have a higher percentage of time-critical conditions, a thorough assessment is important in this subset. A separate triage system has been proposed for the elderly due to the significantly lower sensitivity for this already vulnerable patient group [49]. Access to past medical records at the scene may aid the EMS nurse in patient assessment, and it has been reported as essential to include a physician consultation for non-transport decisions [33]. An increased set of instruments for the EMS nurse is proposed, containing point of care testing, video access for medical consultation, and a digitalised decision support system targeted at the EMS setting, including medical history with machine learning capabilities for assessments of people of all ages with specific cut-offs for VS.

It is obvious that starting the triage process early in the prehospital setting in order to assess the patient to the most appropriate level of care is a new strategy that is developing internationally at different paces. This strategy has an enormous potential. However, important questions such as patient safety and consensus of quality indicators and outcome measures for non-transported patients is still unresolved and thereby highlights the need for improved decision support tools.

### Strengths and limitations

The main strength of this study is that all records were manually reviewed from a relatively large cohort in a systematic fashion. The chief limitation is that the data were collected from a single site in an urban setting with short transportation times to the ED, which may have influenced decisions relating to transport to hospital. Given the limited access to PC, the EMS nurse may have transported the patient in any case; however, it may be problematic to generalise our results outside urban areas. Furthermore, data were collected from EMS patient records and thus there is a dependency on the data recorded by the EMS staff. This is a potential source of bias. In order to maximise data quality, education and information meetings with the EMS staff took place beforehand. Moreover, a selection bias may be present due to the consecutive data collection from the first 1000 assignments each month as events may occur at specific time frames. However, the included data cover 11.4% of the total primary assignments in a year and have been collected each month to capture various fluctuations of certain patient presentations during the year.

## Conclusions

This study concludes that age, sex, past medical history, and type of presentation all appear to influence the EMS assessment process. A number of the patients assessed and transported to the ED by an ALS ambulance could be handled by a physician at a lower level of care and with another type of transport. A small proportion of the non-transported patients were later diagnosed with a time-critical condition; this calls for improved assessment tools at the scene and education focusing on the elderly population in particular. Our results may be useful in addressing resource allocation issues and EMS policies aiming at increased patient safety. This study may serve as a reference for future studies of EMS patient assessments.

## Supplementary information

**Additional file 1.** Definitions of time-critical diagnosis and deviating vital signs according to RETTS-A orange and red triage level.

## Data Availability

The datasets analysed during the current study are available from the corresponding author in response to a reasonable request.

## Abbreviations

ALS: Advanced lifesaving
CTAS: Canadian triage and acuity scale
DEPT: Danish emergency process triage
DMI: Dispatch medical index
ECG: Electrocardiogram
ED: Emergency department
ED72: Emergency department admission within 72 h
EMS: Emergency medical services
ESI: Emergency severity index
ESS: Emergency signs and symptoms
GCS: Glasgow Coma Scale
IAH: Initially assessed as requiring transport to hospital
ICD-10: International Statistical Classification of Diseases and Related Health Problems – 10th revision
ICU: Intensive care unit
MTS: Manchester triage system
PA: Physician assistant
PC: Primary care
Q1: First quartile
Q2: Second quartile
Q3: Third quartile
Q4: Fourth quartile
RETTS-A: Rapid emergency triage and treatment system for adults
UK: United kingdom
VS: Vital signs
