# Supplementary material for: Patient characteristics, triage utilisation, level of care, and outcomes in an unselected adult patient population seen by the emergency medical services: a prospective observational study
Source: BMC Emerg Med. 2020 Jan 30;20:7. doi: 10.1186/s12873-020-0302-x (PMC6993445; doi:10.1186/s12873-020-0302-x)
Supplement: Supplementary file 1 — Additional file 1. Definitions of time-critical diagnosis and deviating vital signs according to RETTS-A orange and red triage level. [file 12873_2020_302_MOESM1_ESM.docx]

| Medical | Traumatic injuries |
| --- | --- |
|  |  |
| Aortic rupture | Cardiac contusion |
| Aortic dissection | Cardiac tamponade |
| Any form of shock | Diaphragm rupture |
| Cardiac arrest | Esophageal rupture |
| Failing heart conducting system | Flail chest |
| Heart failure including pulmonary edema | High energy trauma |
| Intoxication | Massive heamothorax |
| Myocardial infarction | Obstructive airway |
| Pulmonary embolism | Open pneumothorax |
| Septicemia | Pulmonary contusion |
| Tia/Stroke | Tension pneumothorax |
| Unconsciousness | Thoracic aortic rupture/dissection |
| Unstable angina pectoris | Tracheobronchial rupture |

Additional file 1: Table S1

Definition of time-critical conditions for adults

Hagiwara, MA., Nilsson, L., Strömsöe, A., Axelsson, C., Kängström, A. & Herlitz, J. (2016). Patient safety and patient assessment in pre-hospital care: a study protocol. *Scand J Trauma Resusc Emerg Med*. doi: 10.1186/s13049-016-0206-7.

Yamamoto, L. Schoreder, C. Morley, D. & Beliveau, C. (2005). Thoracic trauma: the deadly dozen. Crit Care Nurs Q. 28(1), 22-40.

Deviating vital signs Red/Orange level according to RETTS-A (2016 version)

| A | Obstructive airway | Threat to airway |
| --- | --- | --- |
| B | Respiratory rate > 30 /min  Respiratory rate < 8 /min  Oxygen saturation with supplemental oxygen < 90% | Respiratory rate > 25 /min  Oxygen saturation < 90% |
| C | Pulse rate > 130 /min  Irregular pulse rate > 150 /min  Systolic blood pressure < 90 mm/Hg | Pulse rate > 120 /min  Pulse rate < 40 /min  Diastolic blood pressure > 140 mm/Hg* |
| D | Ongoing seizures  RLS ≥ 4  GCS ≤ 9 | Somnolence  RLS 2-3  GCS 10-12 |
| E |  | Temperature < 35 or > 41 °C |

* Repeated measurements (embedded in ESS); RLS: reaction level scale; GCS: Glasgow coma scale

Predicare AB (www.predicare.se)
